# Optimizing perinatal wellbeing in pregnancy with obesity: a clinical trial with a multi-component nutrition intervention for prevention of gestational diabetes and infant growth and neurodevelopment impairment

**DOI:** 10.3389/fmed.2024.1339428

**Published:** 2024-04-12

**Authors:** Otilia Perichart-Perera, Enrique Reyes-Muñoz, Hector Borboa-Olivares, Ameyalli M. Rodríguez-Cano, Juan Mario Solis Paredes, Larissa Hernández-Hernández, Carolina Rodríguez-Hernández, Isabel González-Ludlow, Blanca V. Suárez-Rico, Maribel Sánchez-Martínez, Ursula Torres-Herrera, Arturo Alejandro Canul-Euan, Maricruz Tolentino-Dolores, Aurora Espejel-Nuñez, Guadalupe Estrada-Gutierrez

**Affiliations:** ^1^Nutrition and Bioprogramming Coordination, Instituto Nacional de Perinatología, Mexico City, Mexico; ^2^Gynecological and Perinatal Endocrinology Coordination, Instituto Nacional de Perinatología, Mexico City, Mexico; ^3^Community Interventions Research Branch, Instituto Nacional de Perinatología, Mexico City, Mexico; ^4^Department of Research in Reproductive and Perinatal Health, Instituto Nacional de Perinatología, Mexico City, Mexico; ^5^Research Division, Instituto Nacional de Perinatología, Mexico City, Mexico; ^6^Department of Immunobiochemistry, Instituto Nacional de Perinatología, Mexico City, Mexico; ^7^Department of Obstetrics, Instituto Nacional de Perinatología, Mexico City, Mexico; ^8^Department of Neurodevelopmental Biology, Instituto Nacional de Perinatología, Mexico City, Mexico

**Keywords:** fetal programming, supplementation, myo-inositol, omega-3, vitamin D, prenatal care, medical nutrition therapy, high-risk pregnancies

## Abstract

Pregnancy complicated by obesity represents an increased risk of unfavorable perinatal outcomes such as gestational diabetes mellitus (GDM), hypertensive disorders in pregnancy, preterm birth, and impaired fetal growth, among others. Obesity is associated with deficiencies of micronutrients, and pregnant women with obesity may have higher needs. The intrauterine environment in pregnancies complicated with obesity is characterized by inflammation and oxidative stress, where maternal nutrition and metabolic status have significant influence and are critical in maternal health and in fetal programming of health in the offspring later in life. Comprehensive lifestyle interventions, including intensive nutrition care, are associated with a lower risk of adverse perinatal outcomes. Routine supplementation during pregnancy includes folic acid and iron; other nutrient supplementation is recommended for high-risk women or women in low-middle income countries. This study is an open label randomized clinical trial of parallel groups (UMIN Clinical Trials Registry: UMIN000052753, https://center6.umin.ac.jp/cgi-open-bin/ctr_e/ctr_view.cgi?recptno=R000060194) to evaluate the effect of an intensive nutrition therapy and nutrient supplementation intervention (folic acid, iron, vitamin D, omega 3 fatty acids, myo-inositol and micronutrients) in pregnant women with obesity on the prevention of GDM, other perinatal outcomes, maternal and newborn nutritional status, and infant growth, adiposity, and neurodevelopment compared to usual care. Given the absence of established nutritional guidelines for managing obesity during pregnancy, there is a pressing need to develop and implement new nutritional programs to enhance perinatal outcomes.

## 1 Introduction

Obesity is a world-wide public health challenge; 18% of women present this condition in 2020 (1). In Mexico, 40% of women in adulthood present this condition (2). Pregnancy complicated by obesity represents an increased risk of unfavorable perinatal outcomes, such as gestational diabetes mellitus (GDM), hypertensive disorders in pregnancy, preterm birth, fetal and neonatal death, abnormal fetal growth, and higher intrapartum complications (3, 4). The intrauterine environment in an obesity-complicated pregnancy is prone to inflammation, oxidative stress, and metabolic disorders that impair nutrient transfer and utilization (5). Obesity is a paradoxical state of malnutrition, where deficiencies of individual microelements may be present (regardless of excessive consumption of calories), in addition to the altered body composition that may influence further nutritional imbalances (6, 7). These nutritional and metabolic derangements during gestation have a profound influence in the metabolic programming of the offspring, leading to a higher risk of obesity and other metabolic complications later in life (8).

GDM risk increases 6-fold in pregnancies with obesity compared to their normal-weight counterparts; women with normal weight had a GDM incidence of 6.1%, increasing to 9.7% and 16.6% in class I and III obesity, respectively (9). Obesity also increases the risk of preeclampsia (RR: 2.07, 95% CI: 1.72 to 2.49, *p* = 0.015) (10). In Mexican women with obesity the risk of GDM increased four times and three times for preeclampsia compared to normal-weight women (11). Pregnant women with obesity have also a higher risk of exceeding the weight gain recommendations. Excessive gestational weight gain has been associated mainly with a higher risk of GDM and C-section, and in some studies with fetal macrosomia, and intrapartum complications (12). In the metanalysis by Dai et al. (13), pre-pregnancy obesity increased the probability of macrosomia in the newborn (AOR = 1.93, 95% CI: 1.65 to 2.27). Macrosomia may increase the risk of intrapartum complications, including shoulder dystocia, perineal lacerations, and other lesions (14). Additionally, there is some evidence about the risk of higher fat mass in the offspring of mothers with obesity, both at birth and during childhood and adolescence (15–17).

Neurodevelopmental alterations in children have also been documented as complications of pregnancies with obesity. In a systematic review (36 cohort studies), a higher risk of attention deficit disorder, autism, developmental delay, and/or emotional and behavioral problems was observed in infants born from women with pregestational overweight or obesity (18).

Derived from the elevated risk of maternal-infant complications linked to obesity during pregnancy, lifestyle interventions, with diet and physical activity components, have been designed and implemented in these women. In a systematic review, there was a possible reduction in GDM risk in the intervention group (diet and exercise) (RR 0.85, 95% CI: 0.71 to 1.01; *p* = 0,07) and a lower gestational weight (MD: −0.89 kg, 95% CI: −1,39 to −0,40) (19) compared to the usual care group. However, studies have failed to demonstrate effectiveness in multiple perinatal outcomes or adiposity later in life.

Different international clinical associations currently recommend medical nutrition therapy (MNT), which consists of intensive nutrition care with a counseling approach to promote behavioral changes to treat obesity, diabetes mellitus, and GDM (20). MNT should be offered by an experienced clinical nutritionist and includes a thorough nutrition assessment, detection of main nutrition problems, design and implementation of a patient-centered nutrition intervention, and the use of various behavioral strategies to promote adherence to changes in food behaviors (21). Unfortunately, it is challenging to differentiate intensive lifestyle interventions (with strong behavioral components) from general diet recommendations or education. Promoting changes to achieve a healthy and sustainable dietary pattern in pregnant women with obesity and other metabolic diseases should be a priority. Healthy dietary patterns, such as Mediterranean and Dietary Approaches to Stop Hypertension (DASH) diet, are related with a protective effect for GDM and hypertensive disorders in pregnancy (22–24). A lower z-score in body mass index (BMI) for age in the first 18 years of life was associated to a high-quality maternal diet (lower inflammatory dietary index or higher Mediterranean diet score) (25).

Nutrient supplementation is one of the main policies to protect vulnerable populations, including pregnant women, newborns, and children, from malnutrition and micronutrient deficiencies (26). The WHO recommendation is a gestational supplementation of folic acid (400 mcg/d, ideally before conception) and iron (30 mg/d) for all women for the prevention of maternal anemia, puerperal sepsis and infant neural tube defects, low birth weight (LBW), and prematurity (27). Calcium supplementation (≥1000 mg/d) is recommended for preeclampsia prevention, mainly for women with an elevated risk and in those whose calcium intake is low (28). The supplementation with multiple micronutrients was included as a routine nutrition intervention for high-risk women or women in low-middle income countries considering the consistent observed effect on the prevention of LBW, small for gestational age (SGA) and premature labor (29, 30).

Pregestational obesity may lead to a higher requirement of some micronutrients, such as vitamin D, omega 3 fatty acids (primarily docosahexaenoic acid-DHA, eicosapentaenoic acid-EPA), and myo-inositol. Routine vitamin D supplementation remains controversial in pregnancy. A Cochrane review reported a lower risk of preeclampsia, GDM, and LBW in women who received vitamin D (31). Reviews of studies showed an association of vitamin D with higher birthweight and length and lower risk of LBW or SGA (32). Other meta-analyses have shown a null effect on preeclampsia with the supplementation of the vitamin. When considering a dose >600 IU/d, compared to ≤600 IU/d, a reduction of GDM and C-section was observed (33). The decision should be individualized, considering risk factors for deficiency (such as obesity), a documented deficiency (25 hydroxyvitamin D −25-OH-D– <20 ng/ml) or insufficiency (25-OH-D ≥20 ng/ml and <30 ng/ml) (34).

The essential fatty acids, DHA and EPA, are relevant in many perinatal processes. DHA is critical for fetal brain development and visual function (35). Considering women with obesity have lower DHA concentrations in the middle of pregnancy or the third trimester (36) and that intake in pregnant women in Mexico is deficient (<100 mg/d) (37), it seems appropriate to supplement DHA and EPA at recommended intake doses. In addition, reviews of studies of gestational supplementation with omega 3 (mainly DHA, EPA), have consistently documented a positive effect on birthweight, preterm birth, LBW or SGA (38). More recent reviews report a lower risk of preeclampsia with the fatty acid supplementation, coming from high-certainty evidence (39). Myo-inositol supplementation could be effective in preventing GDM, hypertensive disorders of pregnancy, and preterm birth (40). A recent meta-analysis (four randomized clinical trials-RCTs) showed a decreased GDM risk (OR 0.32, 95% CI: 0.21 to 0.48; moderate-certainty evidence) and lower glucose values in the oral glucose tolerance test (-OGTT- fasting, 1 h, and 2 h post load) (41) with the gestational supplementation of myo-inositol in women with obesity.

Early nutrition interventions are affordable, safe, well-accepted, and generally cost-effective, and represent a particular chance to prevent obesity and related health problems. The possibility of preventing negative perinatal results and improving fetal growth and nutritional status of this high-risk group, by recommending a healthy dietary pattern and an individualized nutrient supplementation scheme, is an opportunity to decrease the risks of diseases in the future. The present study aims to assess the effect of a multi-component nutrition intervention (intensive nutrition counseling and multiple nutrient supplementation) for women with pregestational obesity during pregnancy, examining its influence on the risk of GDM and other perinatal outcomes, mother-newborn nutritional status, and infant growth, adiposity, and neurodevelopment.

## 2 Methods and analysis

### 2.1 Design

We propose an open-label randomized clinical trial (Table 1) (42) to evaluate an intensive nutrition therapy and supplementation intervention within the prospective cohort OBESO (by its acronym in Spanish “Origen Bioquímico y Epigenético del Sobrepeso y la Obesidad”), in a group of pregnant women with obesity. This protocol was submitted for evaluation by the Ethics and Research Committees within our institution (Provisional number: 2023–1-5) and in the UMIN Clinical Trials Registry (UMIN000052753). All procedures will be conducted according to the Helsinki Declaration. The OBESO cohort (Instituto Nacional de Perinatología –INPer–, Mexico City, since 2017) follows women from the first trimester of pregnancy and through the first two years of their infant's life, and its main aim is to study different factors (biochemical, clinical, lifestyle, and epigenetic) influencing obesity. Pregnant women between 11 and 13.6 weeks of gestation are recruited at the Maternal-Fetal Medicine department.

**Table 1:** Description of blinding in the study (42).

**Group or individual blinded**	**Information withheld**	**Method of blinding**
**Required fields to be completed for all trials described as blinded**		
Person assigning participants to groups	Group assignment	Darkened and sealed envelopes
Participants	None	None
Care providers	None	None
Data collectors and managers	None	None
Outcome assessors	Purpose of study; group assignment; participant characteristics	Participants given numerical identifiers
Statisticians	Participant and group identities	Participants and groups given numerical identifiers
**Supplemental fields for all blinded groups or individuals not mentioned above**		
Laboratory technicians	Group assignment, participant identities	Participants and groups with given numerical identifiers
Outcome adjudicators	Group assignment, participant identities	Participants and groups with given numerical identifiers
Data monitoring (research and ethics committees)	Participant identities	Participants given numerical identifiers
Manuscript writers	Participant identities	Participants given numerical identifiers
	None	None

We used Precis-2 in the design, a 10 domain tool to disclose individual explanatory vs. pragmatic components in our trial (43). The mean score for this study is 2.7 (Supplementary Table).

### 2.2 Selection and treatment of subjects

For this study, all OBESO participants will be invited to participate. Each woman will decide voluntarily whether to participate in the project, without this having any impact on their current care. The signing of the written informed consent will be required.

Women will be selected following the inclusion criteria: adult women without previous diseases, pregnancies with only one fetus (no congenital malformations), and pregestational BMI (pBMI) ≥30 (using pregestational weight -self-reported- and height -measured with a digital stadiometer-). Women will be excluded if they have pregestational diabetes mellitus, chronic hypertension, heart, kidney, liver or autoimmune disease, uncontrolled hypothyroidism, history of bariatric surgery, or if take medications influencing the metabolism of the endocrine system (insulin, metformin, and/or corticosteroids). All women will have an oral glucose tolerance test (OGTT) at the first prenatal care visit, according to Institutional GDM screening procedures. Women with 2 or more altered values or those classified with pregestational diabetes mellitus will be excluded.

#### 2.2.1 Sample size

To define the sample size, the calculation was based on finding a minimal detectable difference of 2% in the incidence of GDM (primary outcome) between the two study groups, with an expected absolute difference of 20% in the incidences of GDM in the control group (30%) and intervention group (10%) as reported in a previous trial evaluation the effect of myo-inositol supplementation on GDM prevention in obese women (44). Using the website https://riskcalc.org/samplesize (45), and considering a superiority trial with a dichotomous outcome (GDM incidence: control group 30% and intervention group 10%), an alpha of 0.05, a statistical power of 90%, and a 20% drop-out rate, we will study 136 women in total (68 participants per group).

Considering the current number of patients recruited in the OBESO cohort, the recruitment phase of this study will end in approximately 24 months. The last follow-up is expected 12 months after the last recruitment (Table 2).

**Table 2:** Timeline.

**Trimester**	**1**	**2**	**3**	**4**	**5**	**6**	**7**	**8**	**9**	**10**	**11**	**12**	**13**	**14**	**15**	**16**	**17**	**18**
Submission and approval of research/ ethics committees	X	X																
Recruitment			X	X	X	X	X	X	X	X								
Prenatal follow-up			X	X	X	X	X	X	X	X	X	X						
Postnatal follow-up					X	X	X	X	X	X	X	X	X	X				
Data registry			X	X	X	X	X	X	X	X	X	X	X	X	X	X		
Analysis of results												X	X	X	X	X	X	X
Writing reports and articles															X	X	X	X

#### 2.2.2 Randomization and allocation

During the first visit, all participants will be randomized (Simple randomization: with a list of random numbers and files numbered sequentially) to one of two groups (parallel design): Group 1-Nutrition intervention, Group 2-Control group (usual prenatal care). Allocation will be performed with envelopes (darkened and sealed) which will have the assigned study group. Once women agree to participate, an external researcher will retrieve the appropriate envelope containing the group assignment. Recruitment and randomization will be performed by clinical staff from the OBESO cohort, who will not be involved with the intervention proposed.

### 2.3 Interventional methods

#### 2.3.1 Medical nutrition therapy and supplementation

*Group 1 (intervention group)*: Women in this group will receive medical prenatal care by an assigned attending obstetrician who will follow institutional guidelines and intensive MNT by a clinical nutritionist.

Intensive MNT will be offered every 4 weeks until the end of pregnancy. According to the nutrition assessment, an initial diet prescription will be calculated, considering intake, metabolic and clinical status, and fetal growth. Initial energy requirements will be estimated with the Mifflin et al. prediction equation using pregestational weight (46). Protein requirements will be estimated as 0.8 g/kg (using weight before pregnancy) (47). No extra energy or protein will be added in the first trimester; for the following trimesters (2nd and 3rd), an additional 360 kcal/d and 475 kcal/d of energy (48) and 9 g/d and 31 g/d of protein (47), respectively, will be considered. Regarding macronutrients, carbohydrates will contribute to total energy with 45%−50%, while lipids with 25%−35%, emphasizing a consumption of saturated fat <7%, and increasing monounsaturated and polyunsaturated fat (specifically omega 3) intake (49). Nutrition goals will be to encourage the adoption of a healthy dietary pattern throughout pregnancy and achieve adequate micronutrient intakes, to promote optimal metabolic control, and to achieve optimal fetal growth.

A healthy eating pattern is characterized by being abundant in vegetables, fruits, whole grains, and low refined cereals and legumes; includes fatty-fish, seeds, nuts, white meat, eggs and low-fat dairy products in moderation; and limits red meat, processed-meat products, added sugars, and ultra-processed products (24, 50). A traditional Mexican dietary pattern will be promoted, recommending culturally accepted local fresh foods and traditional dishes. An individual food plan will be prescribed according to assessed food intake and socio-cultural context. In each visit, strategies such as “goal-setting” and “problem-solving” will be implemented to promote healthy behaviors and adherence to diet recommendations (51). Nutrition intervention will be delivered in the context of intensive counseling, with a strong education component. Educational main themes will include “My Healthy Eating Plate” in pregnancy, adapted from the Harvard Healthy Eating Plate (which includes Mexican, traditional, and unprocessed foods), smart food choices from each group (high-quality carbohydrates and fats), estimating appropriate portion size, the importance of a healthy diet on later health, among others. Breastfeeding education and promotion will be carried out.

In each MNT visit, the dietitian will inquire women about their perception of adherence to dietary recommendations based on a scale of 0%−100%. Barriers and motivators to follow dietary recommendations will be discussed and considered for individual dietary strategies.

Intensive multiple-nutrient supplementation will be recommended to all women in the intervention group. Daily nutrient doses are presented in Table 3, as well as the contribution to daily intake recommendations. This supplementation scheme represents taking 4 capsules daily: 1 multivitamin capsule, 1 folic acid-iron capsule, and 2 myo-inositol-folic acid capsules. Two capsules of vitamin D3 will be taken weekly. A specific pill container (30 days, with AM and PM divisions) will be given to each woman. Counted pills will be distributed in the containers and will be reviewed in the follow-up visit to evaluate adherence in taking the supplements.

**Table 3:** Nutrient supplementation scheme for pregnant women with obesity in the intervention group.

**Nutrient**	**Multivitamin doses**	**Single nutrient supplement doses**	**Total doses**	**% of recommended intake (IOM)**
Folic Acid	400 mcg	Fe+folic acid:200 mcg Myo-inositol+folic acid: 400 mcg	1000 mcg	166%
Iron	14 mg	15 mg	29 mg	107%
Zinc	10 mg		10 mg	91%
Vitamin D3		11,200 IU/week (1600 IU/d)	1600 IU/d	400%
Vitamin C	40 mg		40 mg	47%
Vitamin E	12 mg		12 mg	80%
Thiamine (B1)	1.1 mg		1.1 mg	79%
Riboflavin (B2)	1.4 mg		1.4 mg	100%
Niacin (B3)	16 mg		16 mg	89%
Pantothenic acid (B5)	6 mg		6 mg	100%
Pyridoxin (B6)	1.4 mg		1.4 mg	74%
Biotin (B8)	50 μg		50 μg	166%
Cyanocobalamin (B12)	2.5 μg		2.5 μg	96%
Omega 3 (DHA)	250 mg		250 mg	Approx.100%
Omega 3 (EPA)	50 mg		50 mg	
Manganese	1 mg		1 mg	50%
Copper	1 mg		1 mg	100%
Iodine	0.15 mg		0.15 mg	68%
Selenium	55 μg		55 μg	92%
Myo-inositol		1200 mg 600 mg (2x day)	1200 mg	NA

Institute of Medicine (U.S.A). Dietary Reference Intakes (52).

Women with obesity have high risk of folate deficiency; 1000 mcg/d of folic acid has been suggested during pregnancy to prevent folate deficiency or neural tube disease (53). The rationale for the high doses of vitamin D3 is the elevated proportion of women with deficiency/insufficiency reported in our population. In 2020, we observed that 37% of women in our institution in the first trimester of pregnancy were vitamin D deficient (<20 ng/ml), and only 20% had adequate status (>30 ng/ml). Even when 76% received supplementation during usual prenatal care, 20% of women were still deficient in the third trimester. Prescribed doses were generally low (range: 0–800IU/d), only 10% of women received ≥500IU/day (54). At the National level, 37% of women have vitamin D deficiency in Mexico (55), and obesity is associated with a higher risk of presenting a deficiency in vitamin D. Considering the evidence, controversy exists about doses of vitamin D3 in pregnancy. A Cochrane review showed that doses >600 IU/d of vitamin D3 may be related to a decreased GDM risk, with moderate certainty evidence. Doses up to 4000 IU/d does not seem to elevate the frequency of adverse events (33). The most used doses of myo-inositol are 2g, two times a day (sachets) or its equivalence in oral soft gel capsules (600 mg, twice a day) (56, 57).

Calcium supplementation is also recommended for the prevention of preeclampsia in women at high risk of developing this condition or with low calcium intake (28). Women in this study will have a higher risk of preeclampsia, but calcium supplementation will not be routinely recommended for this group. The obstetrician will decide if calcium supplementation is needed and the individualized doses (minimum 1 g/d).

#### 2.3.2 Control group

*Group 2: control group (usual care):* Participants within this group will be provided with identical medical prenatal care as the intervention group, offered by the same obstetrician, following institutional guidelines. Nutrient supplementation in this group will consist in a multivitamin containing folic acid (400 mcg/d), iron ( ≤30 mg/d), vitamin D (≤400 UI/d), and other micronutrients (meeting ≤100% of recommended intake). In this group omega 3 fatty acids and myo-inositol will not be supplemented. Calcium supplementation (1–2 g/d) will be recommended in some women (as in the intervention group). The attending obstetrician will prescribe this supplementation scheme.

#### 2.3.3 Prenatal medical care (both groups)

The schedule for prenatal care appointments will include monthly visits from the first visit (11–13.6 weeks) until the 32nd week of pregnancy, bi-weekly visits from the 33rd to the 36th week, and weekly appointments from the 36th to the 40th week of gestation. During pregnancy, the risk, and the presence of fetal malformations, chromosomopathies, preeclampsia, premature labor, intrauterine growth restriction (IUGR) and fetal growth will be evaluated according to maternal fetal medicine ultrasound assessments in each trimester. Complete blood count, blood chemistry, urine tests and vaginal swabs will be performed in each trimester or at a higher frequency based on clinical necessity. Blood pressure will be measured in the morning in each visit with an ambulatory device (Spacelabs Healthcare, USA). If the obstetrician in charge considers that a patient has a high risk of preeclampsia, 100 mg of aspirin will be indicated. If a patient is detected with a high risk of preterm birth, 200 mg/d of progesterone will be indicated. The obstetrician will determine the delivery method based on the clinical obstetric background of participants.

### 2.4 Data analysis

#### 2.4.1 Study outcomes

As part of the OBESO cohort, women in both groups will be evaluated in the first (11–13.6 weeks), second (19–24 weeks), and third trimester (28–36 weeks) of gestation. Maternal anthropometric, dietary, and lifestyle data collection will be performed. Fasting blood samples will be collected each visit using Vacutainer tubes (Becton-Dickinson, Franklin Lakes, NJ, USA) and then proceed to centrifugation (3200 rpm, 10 min). Plasma and serum samples will be preserved at −80°C until the time of performing assays, according to the OBESO procedures. A complete blood count will also be performed in each visit. Women will be evaluated at 1 and 6 months after pregnancy, and infants at birth and 1 and 6 months of life (Table 4).

**Table 4:** Schedule of enrolment, intervention, and maternal and infant assessment (58).

	**Study period**							
	**Enrolment**	**Allocation**	**Post-allocation**					
**Timepoint**			**Prenatal**			**Postnatal**		
			**First trimester**	**Second trimester**	**Third trimester**	**Birth**	**1 Month**	**6 Months**
**Enrolment**								
Eligibility screen	X							
Informed consent	X							
Allocation		X						
**Interventions**								
*Intervention group*			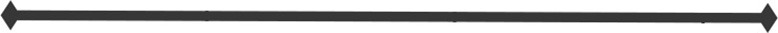					
*Control group*			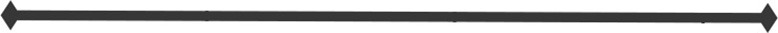					
**Assessments**								
**Baseline variables**								
Pregestational BMI			X					
Sociodemographic characteristics			X					
Clinical and gynecobstetrics data			X					
**Outcome variables**								
**Pregnancy outcomes**								
Fetal/Neonatal death			X	X	X	X	X	
Preterm Birth						X		
Intrauterine growth restriction				X	X			
Delivery						X		
**Maternal outcomes**								
Gestational Diabetes				X	X			
Preeclampsia				X	X			
Gestational weight gain			X	X	X			
Gestational weight gain rate				X	X			
Lipid profile			X		X		X	X
Adipokines			X		X		X	X
Fasting glucose			X		X		X	X
Insulin			X		X			
HOMA-IR			X		X			
Lipopolysaccharide			X		X			
C Reactive Protein			X		X			
Vitamin D			X		X	X	X	X
Complete Blood Count			X	X	X	X	X	X
**Infant outcomes**								
Weight						X	X	X
Small for gestational age						X		
Large for gestational age						X		
Length						X	X	X
Length for age						X	X	X
BMI for age						X	X	X
Head Circumference						X	X	X
Arm Circumference							X	X
Abdominal Circumference						X	X	X
Fat mass						X	X	X
Skinfold thickness							X	X
Brazelton Test							X	
Bailey III Test								X
**Control variables**								
Energy Intake			X	X	X		X	X
Macronutrient intake			X	X	X		X	X
Diet quality			X	X	X		X	X
Ultra-processed food intake			X	X	X		X	X
Physical Activity			X	X	X			
Sleep quality			X		X			
Adherence to Treatment (TMN)			X	X	X			
Adherence to Treatment (self-perceived)			X	X	X			
Adherence to supplementation			X	X	X			

#### 2.4.2 Primary outcome: gestational diabetes mellitus

For this study, the primary outcome is to compare GDM incidence in both groups. For this, a 75 g 2-h OGTT will be performed at 24–28 weeks of gestation. GDM will be stablished if at least one glucose value is altered: fasting ≥ 92 mg/dL, 1-h ≥ 180 mg/dL and 2-h ≥ 153 mg/dL, according to the International Association of Diabetes and Pregnancy Study Groups criteria (59).

All women (in both groups) who develop GDM will be referred to the Institutional Diabetes in Pregnancy Program for multidisciplinary care, including endocrinology treatment and intensive MNT.

#### 2.4.3 Secondary outcomes

##### 2.4.3.1 Adverse perinatal outcomes

- Preeclampsia: Preeclampsia will be considered if a woman with previously normal blood pressure presents hypertension, defined as a value ≥140 mmHg in systolic pressure and/or 90 mmHg in diastolic pressure. This finding must occur on two occasions (minimum 4 h apart) after the 20th week of pregnancy. Hypertension must be combined with the presence of proteinuria, which can be evaluated with urine protein (≥300 mg/24h), with the protein/creatinine ratio (≥0.3 mg/dL) or with a dipstick (reading of 2+) (60).
- Preterm birth: Live birth before 37 weeks of gestation. The first-trimester ultrasound measurement will be the basis for establishing gestational age.
- Fetal and neonatal death: The death of a fetus *in utero* at any stage of pregnancy and the death at birth or within the first 28 days after birth, respectively.
- Mode of delivery: Either vaginal birth or cesarean delivery.
- Fetal growth alterations: Fetal growth will be evaluated by ultrasound, measuring biparietal diameter, circumferences of the head and the abdomen, and the length of the femur and the humerus. The Hadlock formula (preloaded in the ultrasound machines) will be used to estimate fetal weight. The growth percentile will be assigned according to the Hospital Clinic de Barcelona calculator (Calculadora v2021 in Spanish, https://portal.medicinafetalbarcelona.org/calc/), considering gestational age and fetal gender. Fetuses with an estimated fetal weight <10th percentile with alterations in the pulsatility index of various arteries (umbilical, middle cerebral, ductus venosus, aortic isthmus, uterine), or those fetuses with weight <3rd percentile regardless of hemodynamic alterations will be classified as IUGR. Fetuses classified as SGA will be those between the 3rd−10th percentile of estimated fetal weight with normal hemodynamic assessment. Large for gestational age (LGA) classification will be with an estimated fetal weight >90th percentile (61).

##### 2.4.3.2 Maternal nutrition and metabolic status

- Gestational weight gain and postpartum weight retention: In the initial visit, women's height will be measured based on the Lohman's technique (62), with a digital stadiometer (model 264, SECA, Hamburg, Germany) recording measurements to the nearest 0.1 cm. According to the WHO criteria, pBMI will be used to classify normal, overweight, and obesity status (63). For weight measurement (rounded to the nearest ±0.1 kg), at each visit participants will stand on a digital scale (BMB-800, TANITA, Japan), wearing light clothing and no shoes. In the third-trimester visit (28–34.6 weeks) gestational weight gain will be assessed based on the guidelines established by the Institute of Medicine, classifying women with insufficient, adequate, or excessive weight gain, considering gestational age and pBMI (64). Maternal weight retention (kg) will be considered the difference between postpartum and pregestational self-reported weight.
- Metabolic markers and inflammation: Biochemical markers will be assessed in the first- and third-trimester visits. Complete blood count will be measured with the impedance method using a Coulter (ACT-5 diff diluent C8547169, ACT-5 diff WBC Lyse C8547170, ACT-5 diff HGB Lyse C8547168, ACT-5 diff rinse C8547167, ACT-5 diff Fixative C8547171, ACT-5diff Beckman Coulter, USA). Serum total cholesterol (DIA11300910923–2), HDL-cholesterol (DIA13521910920–2), LDL-cholesterol (DIA14121910921–2), triacylglycerides (DIA15710910923–2) and glucose (DIA12500910923–2) will be measured using enzymatic colorimetric methods with an automated analyzer (Response 910, DiaSys Diagnostic Systems GmbH, Germany). Insulin will be measured by chemiluminescence using a commercial kit [sensitivity ≤1.0 uU/mL, coefficient of variation (CV) ≤7%] (B8K4B0, Architect Insulin, Abbott Laboratories, USA). The homeostatic model assessment of insulin resistance (HOMA-IR) will be computed according to the glucose (mg/dL) and insulin (uIU/mL) values (65). Enzyme-linked immunosorbent assays (ELISA) will be employed to quantify serum adiponectin (DY10659), leptin (DY398), and resistin (DY1359) with the use of commercial kits, following the instructions provided by the manufacturer (R&D Systems Inc., Minneapolis, MN, USA). High-sensitivity C-reactive protein, a chronic inflammation indicator linked to fasting insulin, insulin resistance, and metabolic syndrome (66), will be measured by nephelometry (sensitivity 0.8 mg/L, intra-assay precision ≤4%) (P14.03.104311–00; Genrui Biotech, Shenzhen, China). Lipopolysaccharide as marker of metabolic endotoxemia (67), will be measured in serum by ELISA. It will be measured by ELISA (MBS266722; MyBiosource, San Diego, USA) (sensitivity 5 ng/mL, intra-assay precision ≤8%).

##### 2.4.3.3 Neonatal and infant outcomes

Two experienced and trained research nutritionists will perform anthropometric and body composition measurements in the newborn at birth and in the infant at 1 and 6 months. Neurodevelopment assessment will be applied to infants at 1 and 6 months by licensed pediatric psychologists.

- Newborn and infant nutritional status: The newborn's sex will be recorded. Weight will be measured (nearest 0.1 kg) with a pediatric scale (Baby/Mommy 1582, Tanita, Tokyo, Japan) at birth and at subsequent infant visits, with the digital scale integrated into the PEAPOD body composition system (COSMEDUSA Inc, Concord, California). An infantometer (SECA 207, Hamburg, Germany) will be used to measure recumbent length (duplicate measurement, recording the average). Head and abdominal circumferences will be taken with an anthropometric tape (Gulick II, Country Technology, WI, USA). All anthropometric measurements will be performed following Lohman's technique (62). A birthweight < 2500 g and >4000 g will define LBW and macrosomia, respectively. Newborns categorized as SGA or LGA will be identified based on a weight below the 10th percentile or above the 90th percentile for their gestational age, respectively. To assess nutritional status, nutritional indices will be computed and interpreted: weight/age, length/age, weight/length, BMI/age, and head circumference/age. The WHO reference growth criteria will be used for term infants, and the Intergrowth reference (birth and postnatal) for preterm infants.
- Infant body composition: Body density will be measured using an air-displacement plethysmography equipment (PEAPOD, COSMED Inc. USA, California, USA) to obtain infant fat mass (in kg). Prior each test, the PEAPOD system must pass a series of calibrations recommended by the manufacturer. Fat mass estimation consist of placing the infant within the chamber to measure the body volume (wearing no clothes and placing a cap on the hair). Once body density is determined (using weight and body volume measurements), the equipment's software employs the Fomon's equation to calculate fat mass. (68).
- Neurodevelopment outcomes: The Brazelton Neonatal Behavioral Assessment Scale (NBAS) will be performed in the first month of life (using corrected age for preterm babies). Comprising six sub-tests, this assessment will be conducted in the presence of (minimum) one parent, and normally spans a duration of 30–40 min. The NBAS analyzes different development tasks: autonomic and motor function, orientation, range of state and regulation of state. The behavioral items will be converted into percentiles. Classification will be done according to reference curves for Hispanic populations. Abnormal scores will be considered if < 10th percentile (69). The Bayley Scales of Infant Development, third edition (BSID-III) assesses six domains in the infant development: cognitive and socio-emotional skills, receptive and expressive language, fine and gross motor development. This is an individually administered instrument (women fill out the questionnaire) that will be applied (lasts between 15 and 25 minutes) in the 1st and 6th month of the infant's life (70).

##### 2.4.3.4 Control variables

- Dietary intake: The dietary assessment will be performed by a nutrition professional with experience and training in the multiple-pass 24-h recall technique, using food replicas, measuring cups/spoons (standard), among others, as support for a more accurate portion estimation. Each trimester will include two dietary recalls; additional visits (to those in the OBESO cohort) will be programmed to complete 6 recalls. Nutritional analysis will be implemented using the Food Processor SQL software (version 14.0, ESHA Research, Salem, OR, USA), which includes Mexican foods in the database and permits standardized recipes and the inclusion of foods commonly consumed in our population (having as reference the Mexican Tables of Nutritional Value as well as product labels). From the software analysis, the total consumption of energy (kcal), carbohydrates, protein and lipids will be recorded, as well as the consumption of saturated, monounsaturated, polyunsaturated fat, omega 3 and 6 fatty acids (both in grams and as a percentage of the total energy value), and fiber (g). The data of the two dietary recalls within each trimester will be averaged to establish the usual intake. Ultra-processed products will be identified in each recall, using the NOVA definition (71, 72); food labels will be reviewed if needed. Energy intake from all ultra-processed foods consumed will be computed in each trimester as a percentage of the total energy intake.
- Adherence to the intervention: The number of MNT visits and self-reported perceived adherence to dietary recommendations (0–10 scale) will be used to evaluate adherence to MNT.
- Adherence to supplementation: The percentage of pills taken each month (pills taken vs. total pills recommended × 100) will be recorded to evaluate adherence to supplementation. Each woman will receive a 30-day pill container with daily distribution of recommended supplements. The container will be reviewed during each MNT visit.
- Pharmacological treatment: Any medication (corticosteroids, antibiotics, progesterone, aspirin, metformin, insulin, among others) the women take during pregnancy will be recorded.
- Physical activity: The International Physical Activity Questionnaire-Short version (73) will be applied in the first and the last trimester of gestation. The total metabolic equivalent of task (METs) will be quantified per hour/week.
- Sleep quality: The Pittsburgh Sleep Quality Index scale (74) will be applied during the initial and the final trimester of pregnancy. The total score will be recorded, and women will be classified as having good quality sleep or bad quality sleep.

#### 2.4.4 Data analysis

The analyses of this study will adhere to the recommended guidelines outlined in the CONSORT 2010 statement for reporting randomized trials involving parallel groups (75). An intention-to-treat analysis will be performed. The initial homogeneity of the sample will be assessed using either Student's *t*-test or the Mann–Whitney U test, according to the data distribution of the continuous variables. For categorical variables, the X2 test will be performed. The evaluation of the intervention's effect on the incidence of GDM will be conducted using the relative risk and the risk difference and their corresponding 95% confidence interval. For continuous secondary variables, inter-group mean differences will be tested (Student's *t*-test, Mann–Whitney U test, one-way-ANOVA test); for categorical secondary variables, *X*2 and Fisher test will be performed. Mixed models incorporating random and fixed intercept/slope effects will be carried out to estimate the impact of the intervention on postpartum maternal weight and fat mass, and infant BMI and fat mass. Repeated measures ANOVA will be performed to evaluate both the overall and stratified trajectory impact of the intervention on infant growth and adiposity during the first 6 months. Multiple regression models (linear or logistic) will be performed, and the adjusted relative risk obtained to evaluate the effect of the intervention, while considering control variables. For women with a GDM diagnosis, the adjustment will include multidisciplinary treatment variables (endocrinology and MNT visits, insulin/metformin use and/or doses, last glucose value, average last visit capillary blood glucose, among others). The SPSS^®^ software (version 24) will be used to perform the statistical analyses. Missing data will be analyzed and handled appropriately, according to type and percentage of missing data (76).

##### 2.4.4.1 Monitoring

All adverse events that occur from the beginning of the study and until the conclusion of the intervention will be recorded. The intervention with nutrition supplements is considered low-risk and unrelated to severe adverse events. However, the most common complaints of a high-dose iron supplementation regimen are gastrointestinal symptoms, including constipation, nausea, vomiting, and diarrhea (30). Folic acid is likely safe; with minimal reports indicating side effects such as nausea, vomiting, constipation, or diarrhea (77). When taken in higher doses, folic acid supplementation could cause diarrhea, rashes, sleep disorders and could mask vitamin B12 deficiency (78). Hypervitaminosis is rare (79) regarding vitamin D. Prolonged use of high (>4000UI/d) vitamin D doses was associated with a tendency of higher hypercalcemia risk (80). Omega supplementation is usually safe; only mild symptoms were associated with its consumption such as fishy taste, burping, dyspepsia, gastrointestinal discomfort or pain, nausea, diarrhea, and slightly higher tendency of bleeding (81, 82). Nausea, diarrhea, flatus are some of the gastrointestinal side effects reported with a very high myo-inositol dose (12 g/day) (83, 84).

Established procedures of the institutional committees contemplate periodic audits for active research projects, in addition to submitting quarterly reports on the progress and eventualities in the implementation of the protocol.

Monthly evaluations of the adverse effects form of each participant will be performed. If a severe effect is reported, it will be turned to the research and ethics committee for its evaluation. If the stopping of the trial is needed, it will be decided by the aforementioned committees.

## 3 Discussion

While there is increasing evidence showing that gestational obesity and metabolic derangements are risk factors for perinatal complications, and that these conditions have long-term implications for mother and infant health, there are still inconsistencies about what prevention and intervention strategies should be implemented at the clinical level (85). There is a lack of standardized and effective interventions to reduce perinatal risk and improve the metabolic health of the next generation.

In Mexico, as in many other countries, there is a dual challenge of malnutrition. On one hand, metabolic disorders have been increasing in recent years. Forty percent of adult women have obesity (2), starting pregnancy with this condition. GDM and preeclampsia frequency persist in an ongoing upward trajectory (86, 87). Childhood and adolescent obesity prevalence is one of the highest around the world, where 18.1% of school-age children and 17.2% of adolescents have obesity (88), with an alarming increase of 24% from 2016 to 2020–2022 in school-age boys. On the other hand, 35% of pregnant women have anemia (89), and 29% and 31% of women of reproductive age have iron and vitamin D deficiency, respectively (55, 90). In national datasets, it is observed that over 50% of women exhibit inadequate dietary intake of iron, calcium, vitamin A, folate, and vitamin E (91, 92). Mexican pregnant women also reported a deficient intake of DHA (< 100 mg/d) (37).

In addition, pregnant women with obesity frequently lack knowledge about the health consequences of obesity during pregnancy for the mother-infant dyad. In a study done in the US, only 52% of pregnant women received advice about weight gain, 63% about physical activity, and only 56% received nutrition recommendations (93). Communication with health professionals may be experimented as stressful and confusing. Many communication barriers have been reported between patients and health professionals such as lack of time, lack of sensitivity to obesity, weight stigma, negative attitudes, or lack of orientation or practical advice (94). In Mexico, there are no clinical guidelines to manage obesity in pregnancy, and medical nutrition therapy is not integrated into primary prenatal care.

In 2017, our group started an institutional cohort to study early determinants of adiposity, metabolic disorders, and neurodevelopment by studying pregnant women. The Instituto Nacional de Perinatología in Mexico City is a major governmental tertiary hospital in Mexico that offers clinical care to women without social security in the gynecology, obstetrics, reproduction, and neonatology fields. It is also a research (72 researchers) and training hospital (300 students). The OBESO cohort follows-up women during pregnancy and their infants until 2 years old, and lifestyle, dietary, nutrition, metabolic, hormonal, inflammation, oxidative stress, microbiota, and environmental factors are being studied. We have examined 520 women from 2017 to 2023, with different baseline metabolic and clinical risk status. Within this cohort, 27% of women had pregestational obesity, and at the end of pregnancy, one-third of them subsequently presented excessive gestational weight gain. At 3 months of age, 17% of infants were classified as at risk of overweight, and 3% were classified as overweight.

The OBESO cohort is still ongoing and has generated new knowledge on how all these environmental and individual maternal factors influence the risk of developing perinatal complications and their influence on the nutritional status of newborns, adiposity, growth, and neurodevelopment during the infant's first 2 years of life. Prediction models have been created using artificial neural networks with first-trimester maternal anthropometric and biochemical data to predict birth size (95). It has been possible to demonstrate that a high-quality diet during pregnancy protects against LBW and SGA (96) and that a lower intake of ultra-processed products is related with better nutrient intake and higher maternal antioxidant response (97). We were the first group that reported longitudinal data on the status of vitamin D during pregnancy in Latin America, reporting an elevated frequency of deficiency (37% at the beginning of pregnancy) and noting that common prescribed doses (200–400 IU/d) appear to be insufficient in achieving an adequate concentration of vitamin D in the gestational period (54).

The feasibility of this study is supported by the Instituto Nacional de Perinatología, a National Health Institute focused on addressing complex perinatal health issues on a national scale. The institute conducts diverse research—basic, clinical, epidemiological, and sociomedical–to create care models for pregnant women and their infants. Our institute has an annual attendance of 3,500 births and is the regional reference in perinatal medicine, so it is our role to generate knowledge that will help develop an evidence-based clinical practice guideline to provide optimal prenatal care in this high-risk population. Our results will be the basis for designing and implementing new nutrition programs and policies to improve perinatal outcomes and palliate the adverse metabolic and neurodevelopmental programming associated with obesity in pregnancy.

Part of the strengths of this protocol is the experience we have acquired during the last 8 years within the OBESO cohort, in addition to our experience in conducting RCTs with nutrition interventions (98–100). This expertise, combined with our available human resources, specialized experts in various fields, and high-quality measurement equipment, enables us to enroll women and systematically assess various outcomes throughout pregnancy efficiently. Our infrastructure comprises medical facilities for maternal-fetal medicine, specialized nutrition and metabolic laboratories, administrative offices, sample collection, and a neurodevelopment assessment area. Our laboratory participates in an external quality control program, and control samples are sent monthly to evaluate intra-assay variation. Quality control performance is validated each year. We also have a specialized area for maternal nutritional assessment equipped with food replicas for portion estimation, weight scale, stadiometer, as well as a bioelectrical impedance equipment for pregnancy and postpartum follow-up. For newborn evaluation, we have equipment for anthropometric measurement (infantometer, Lange plicometer, anthropometric measuring tape, digital scales), and an air displacement plethysmography (PEAPOD) in the hospital settings. For infant follow-up, we have a body composition area equipped with an additional PEAPOD equipment and a separate set of anthropometric tools in the research facility. Research nutritionist in charge of anthropometrical measurements, are experienced and well-trained professionals who collaborate in international studies. Another strength is the independence among researchers, the clinical and laboratory staff, which may reduce bias toward one study group.

The protocol has also some limitations. Pregnant women receiving prenatal care at our hospital are classified as high-risk. Our strict inclusion criteria, where only women without any diagnosed disease will be included, may be challenging. However, we have been able to recruit women with these characteristics in our cohort. In addition, the measurement of adherence to this intervention is complicated and we included subjective and more objective methodologies, such as quantitative dietary assessment. People with obesity tend to sub-report dietary intake, so this could affect the results. The design of this study does not allow to assess the independent effect of each supplemented nutrient on the study outcomes; however, our aim is to evaluate the global effect of multiple strategies associated with different benefits in pregnancies complicated with obesity. Women in the control group who develop GDM will receive multidisciplinary treatment, which possibly will benefit infant outcomes; however we can statistically measure the effect of treatment by including different variables in the multivariate models. We also recognize that implementing the intervention may prove difficult (low pragmatic attitude), particularly in typical prenatal care settings in regions like Mexico and other low to middle-income countries, where obtaining all necessary supplements, test and ensuring the presence of a nutrition expert on-site could be challenging. On the other hand, the nature of usual prenatal care suggests a higher likelihood of desertion, which could impede the completion and efficacy of the intervention. These limitations underscore the need for careful consideration and adaptation of strategies to address resource constraints and participant adherence challenges in future studies.

Interventions in early stages have a high potential for preventing different health conditions, including obesity, where nutrition strategies stand out for being cost-effective. The proposed multi-component nutrition intervention includes many of the nutrition strategies that have been proven effective in some studies. Our institution is the regional reference in perinatal medicine, so it is our role to generate knowledge that will help develop an evidence-based clinical practice guideline to provide optimal prenatal care for these women prone to preventable perinatal complications. Our results will offer a basis for designing and implementing new nutrition programs and policies to improve perinatal outcomes and palliate adverse metabolic and neurodevelopmental programming.

## Ethics statement

This study was submitted for evaluation and reviewed and approved by the Research and Ethics Committees within our institution (Provisional Number: 2023-1-5). All procedures were conducted according to the Helsinki Declaration. The patients provided their written informed consent to participate in this study.

## Author contributions

OP-P: Conceptualization, Data curation, Formal analysis, Methodology, Supervision, Validation, Visualization, Writing – original draft. ER-M: Formal analysis, Methodology, Project administration, Validation, Writing – review & editing. HB-O: Methodology, Project administration, Validation, Writing – review & editing. AR-C: Data curation, Formal analysis, Funding acquisition, Investigation, Visualization, Writing – review & editing. JS: Data curation, Resources, Writing – review & editing. LH-H: Data curation, Investigation, Visualization, Writing – original draft. CR-H: Data curation, Investigation, Writing – review & editing. IG-L: Data curation, Formal analysis, Investigation, Visualization, Writing – review & editing. BS-R: Data curation, Investigation, Writing – review & editing. MS-M: Investigation, Writing – review & editing. UT-H: Investigation, Supervision, Writing – review & editing. AC-E: Investigation, Supervision, Writing – review & editing. MT-D: Investigation, Methodology, Resources, Writing – review & editing. AE-N: Investigation, Resources, Writing – review & editing. GE-G: Funding acquisition, Methodology, Project administration, Writing – review & editing.
