# Supplementary material for: Optimizing perinatal wellbeing in pregnancy with obesity: a clinical trial with a multi-component nutrition intervention for prevention of gestational diabetes and infant growth and neurodevelopment impairment
Source: Front Med (Lausanne). 2024 Apr 12;11:1339428. doi: 10.3389/fmed.2024.1339428 (PMC11045894; doi:10.3389/fmed.2024.1339428)
Supplement: Supplementary file 1 [file Table_1.DOCX]

Supplementary Material

Optimizing perinatal well-being in pregnancy with obesity: A clinical trial with a multi-component nutrition intervention for prevention of gestational diabetes and infant growth and neurodevelopment impairment

Perichart-Perera O^1^†, Reyes-Muñoz E^2^†, Borboa-Olivares H^3^, Rodríguez-Cano AM^1^, Solis-Paredes M^4^, Hernández-Hernández L^1^, Rodríguez-Hernández C^1^, González-Ludlow I^1^, Suárez-Rico BV^5^, Sánchez-Martínez M^6^, Torres-Herrera U^7^, Canul-Euan A^8^, Tolentino-Dolores MC^1^, Espejel-Nuñez A^6^, Estrada-Gutierrez G^5^*

*** Correspondence:** Estrada-Gutiérrez G, [gpestrad@gmail.com](mailto:gpestrad@gmail.com)

# Supplementary Table. PRECIS-2 scores for trial domains

|  | **Domain** | **Score** | **Rationale** |
| --- | --- | --- | --- |
| 1 | Eligibility Criteria | 4 | Obesity is a very frequent problem in our population, many women may be eligible for this intervention. |
| 2 | Recruitment Path | 3 | Difficult to recruit women in the first trimester of pregnancy in general healthcare settings in Mexico. |
| 3 | Setting | 3 | Some settings may be adequate to implement this intervention, depending on their specific human and material resources. |
| 4 | Organisation intervention | 2 | Difficult to find in usual prenatal care the organization to deliver exactly this intervention. |
| 5 | Flexibility of experimental intervention – Delivery | 2 | May be difficult in usual prenatal care settings in Mexico to have the complete supplements and the nutrition expert on site. |
| 6 | Flexibility of experimental intervention – Adherence | 3 | With the correct intervention delivery, it may be possible to achieve adherence in women in usual prenatal care. |
| 7 | Follow-up | 3 | Although prenatal care usually consists of monthly visits, it may be difficult to guarantee the proposed intensive monitoring in all settings, depending on the human and material resources of each setting. |
| 8 | Outcome | 3 | Relevant to participants. Depends on the setting (rural-urban regions, high-low income), but many hospitals now have implemented GDM diagnosis at 24-28 weeks of gestation. |
| 9 | Analysis | 2 | In usual prenatal care, more desertion could be expected. |

Loudon K, Treweek S, Sullivan F, Donnan P, Thorpe KE, Zwarenstein M. The PRECIS-2 tool: designing trials that are fit for purpose. BMJ. 2015 May 8;350:h2147–h2147. doi: 10.1136/bmj.h2147
